# GluA2 palmitoylation by SELENOK modulates AMPAR assembly and synaptic plasticity in Alzheimer's disease

**DOI:** 10.1016/j.redox.2025.103831

**Published:** 2025-08-21

**Authors:** Jiaying Peng, Zhiyu Cai, Pei Ouyang, Shujing Lin, Shurui Zhang, Danchan Liang, Ziqi Feng, Changbin Chen, Xilin Ye, Guoli Song, Zhonghao Zhang

**Affiliations:** a Brain Disease and Big Data Research Institute, Shenzhen Key Laboratory of Marine Bioresources and Ecology, College of Life Sciences and Oceanography, Shenzhen University, Shenzhen, China; b Shenzhen-Hong Kong Institute of Brain Science-Shenzhen Fundamental Research Institutions, Shenzhen, China

**Keywords:** Selenoprotein K, Synaptic plasticity, Alzheimer's disease, GluA2 palmitoylation, AMPAR assembly

## Abstract

Se is essential for central nervous system function, and its deficiency is strongly associated with cognitive decline, especially in neurodegenerative disorders such as Alzheimer's disease (AD). Although Se exerts its effects through selenoproteins, the molecular basis of its neuroprotective action remains unclear. Among selenoproteins, the endoplasmic reticulum (ER)-resident selenoprotein K (SELENOK) is closely linked to cognitive function and therapeutic potential in AD. Here, we examined how SELENOK regulates synaptic plasticity and contributes to Se-mediated neuroprotection in AD. Using age-gradient SELENOK knockout models and palmitoyl-proteomics, we identified GluA2 (formerly GluR2) as a key downstream target. Mechanistically, SELENOK enhanced the activity of DHHC6, an ER-localized palmitoyltransferase, to promote GluA2 palmitoylation, facilitating its ER retention and efficient assembly of AMPA-type glutamate receptors (AMPARs). Notably, GluA2 palmitoylation was reduced in both AD model mice and postmortem brains of patients with AD. Importantly, neuronal overexpression of SELENOK in the hippocampus restored synaptic plasticity and cognitive function in AD mice. Overall, this study uncovers a novel SELENOK-dependent mechanism regulating AMPAR assembly, offering experimental support for developing Se-based therapeutic strategies for AD.

## 1 Introduction

Se plays a critical role in neurodevelopment and the maintenance of brain structure and function. Its deficiency is strongly associated with cognitive decline and significant impairments in synaptic plasticity [1]. Supplementation with Se effectively enhances synaptic function under both physiological and pathological conditions [2,3], particularly in neurodegenerative diseases such as Alzheimer's disease (AD) [4,5]. Although the antioxidant properties of Se have been extensively studied in relation to its beneficial effects on synaptic proteins and plasticity [6], the underlying mechanisms—particularly those involving Se-dependent selenoproteins—remain poorly understood.

Selenoprotein K (SELENOK), an endoplasmic reticulum (ER)-resident transmembrane protein, is highly expressed in the brain and plays critical roles in regulating ER stress, redox homeostasis, protein metabolism, and intracellular Ca^2+^ balance [7]. Our previous study showed that SELENOK contributes significantly to the anti-AD effects of Se, with brain SELENOK expression directly correlating with AD pathology in vivo [8]. Notably, SELENOK-knockout mice exhibit marked cognitive deficits and impaired synaptic plasticity [4], indicating a strong link between SELENOK and synaptic function. However, the precise molecular mechanisms and downstream targets through which SELENOK regulates synaptic function remain unclear. Elucidating SELENOK's molecular role in synaptic plasticity may, therefore, offer valuable insights into fundamental brain function and potential therapeutic avenues for AD.

Synaptic plasticity, which is essential for learning, memory, and higher cognitive functions, depends primarily on the dynamic behavior of synaptic receptors, including their mobility and assembly [9]. Post-translational modifications, such as phosphorylation, ubiquitination, nitrosylation, and palmitoylation, play key roles in regulating synaptic plasticity [[10], [11], [12], [13]]. Among these modifications, S-palmitoylation—a reversible lipid modification of cysteine residues catalyzed by DHHC-domain-containing S-acyltransferases—regulates protein membrane localization, trafficking, conformational changes, and complex assembly and maturation [14]. Ionotropic glutamate receptors, particularly N-methyl-d-aspartate receptors (NMDARs) and α-amino-3-hydroxy-5-methyl-4-isoxazolepropionic acid receptors (AMPARs), are central to excitatory neurotransmission and synaptic plasticity. Notably, the stability, localization, trafficking, and recycling of these receptors are highly dependent on palmitoylation [15].

Most palmitoyl acyltransferases (DHHCs) associated with synaptic proteins or receptors, such as DHHC2, DHHC3, DHHC5, DHHC7, DHHC8, and DHHC15, are predominantly localized to the Golgi apparatus [16,17]. In contrast, the GluA2 subunit of AMPARs is uniquely palmitoylated within the ER [18]; however, the functional significance of this modification and the specific DHHC enzyme involved remain unclear. SELENOK has been recognized for its role in protein palmitoylation [19], with its SH3 domain mediating interaction with DHHC6 [20], an ER-localized enzyme that regulates the palmitoylation of proteins such as CD36, IP3R, and FcγR [[21], [22], [23]]. Accordingly, the SELENOK–DHHC6 complex is well-positioned, both spatially and temporally, to mediate GluA2 palmitoylation.

Here, we identified the GluA2 subunit as a critical target through which SELENOK regulates synaptic plasticity, with DHHC6 serving as the principal palmitoyl acyltransferase responsible for the GluA2 palmitoylation within the ER. Mechanistically, this modification generates a vital ER-resident pool of GluA2, facilitating efficient AMPAR assembly. Collectively, our findings reveal a novel mechanism linking SELENOK-dependent GluA2 palmitoylation to AMPAR assembly, underscoring its essential role in synaptic plasticity.

## 2 Materials and methods

### 2.1 Mice

C57BL/6 N mice, used as wild-type controls, were obtained from Vital River Laboratories (Beijing, China). SELENOK knockout (Selenok^−/−^, KO) mice were generated and maintained by Biocytogen (Beijing, China). The 5 × FAD transgenic mice (MMRRC_034848-JAX) were purchased from The Jackson Laboratory (Bar Harbor, ME, USA). All animals were housed in groups of 5–6 per cage under standard laboratory conditions (22 ± 2 °C, 12-h light/dark cycle) with ad libitum access to food and water. All animal procedures were approved by the Animal Ethics and Welfare Committee of Shenzhen University (permit number: IACUC-202300052) and were performed in accordance with institutional guidelines.

### 2.2 Human subjects

Postmortem brain tissues were obtained from the Chinese Brain Bank Center at South-Central Minzu University (Wuhan, China), with informed consent obtained from all donors. Detailed demographic information is presented in Table S1. The influence of sex and gender identity on study outcomes was not evaluated because of the limited sample size and scope of the current study.

### 2.3 Cell culture, viral infection, and plasmid transfection

Human embryonic kidney 293 (HEK293) and Neuro-2a (N2a) cells (Fuheng Biology, Shanghai, China) were cultured in DMEM and DMEM/Opti-MEM (1:1) (Thermo Fisher Scientific, Waltham, MA, USA), respectively, both supplemented with 10 % fetal bovine serum (FBS). SELENOK knockout N2a cells were generated using the CRISPR-Cas9 system. Primary hippocampal neurons were isolated from E15–17 mouse embryos, seeded at a density of 0.5 × 10^6^ cells/well on poly-l-lysine-coated 6-well plates, and maintained in neurobasal medium containing 2 % B27, 0.5 mM l-glutamine, and 50 U/mL penicillin-streptomycin. Neurons were used after 12–15 d in vitro.

Adenoviral vectors were constructed by Obio Technology (Shanghai, China). The SELENOK knockdown (shSELK; GGTGGATGAGGAAGGTAAATG) and control (TTCTCCGAACGTGTCACGT) constructs were driven by the CMV promoter. The SELENOK overexpression virus carried a 285 bp coding sequence (CDS) and a 595 bp 3′-untranslated region (UTR; NM_019979.2). For infection, cells were plated in 6-well plates, and after adherence, the medium was replaced with 750 μL of serum-free medium. Virus was added according to the following formula: volume = [cell number × multiplicity of infection (MOI)]/virus titer (MOI = 200). After 2 h, 750 μL of complete medium was added. The medium was replaced within 24 h, and the cells were collected 48 h post-infection.

Full-length cDNAs for human GluA1, GluA2, GluA3, and GluA2 palmitoylation-site mutants were cloned into pcDNA3.1 with C-terminal FLAG tags (General Biology, Anhui, China). Transfections were performed when cells reached 70 %–90 % confluence. Plasmid–reagent complexes were prepared according to the manufacturer's instructions, incubated for 20 min at room temperature, and added dropwise (500 μL/well). The medium was then replaced after 4 h. Fluorescence was measured the following day, and cells were used for experiments after 24 h.

### 2.4 AAV delivery

AAVs (AAV2/B10) encoding SELENOK overexpression (SELENOK-OE; ITR-CAG-Selenok (CDS + UTR)-PGK-EGFP-WPRES-ITR) were packaged by *BrainVTA* (Wuhan, China). For viral delivery into the cortex and hippocampus, 16-week-old mice were anesthetized with an intraperitoneal injection of sodium pentobarbital (80 mg/kg). Once fully anesthetized, 100 μL of AAV solution was administered into each eye via retro-orbital injection at an angle of approximately 30° to the midline, with the needle tip directed toward the medial canthus.

### 2.5 Morris water maze

The maze consisted of a circular opaque pool (160 cm diameter, 50 cm depth) filled with water maintained at 22 °C. A hidden platform was submerged 1–2 cm below the water surface. Visual cues were minimized using surrounding curtains. An overhead camera and Smart v3.0 video tracking system (TECHMAN, Chengdu, China) were used to record and analyze swimming paths. The pool was virtually divided into four equal quadrants, each representing 25 % of the total area. The test lasted 8 days: day 1 involved orientation and habituation training; days 2–5 consisted of acquisition trials to train mice to locate the hidden platform; on days 6 and 8, the platform was removed for conducting 24-h and 72-h probe tests to assess short- and long-term spatial memory, respectively.

### 2.6 Open-field test

The open-field test was conducted to assess spontaneous locomotor activity, exploratory behavior, and anxiety-like responses. The apparatus consisted of a cuboidal chamber (100 cm × 100 cm × 40 cm), with the floor divided into 25 squares by black grid lines. After a 1-min habituation period, each mouse was placed at the center of the arena and allowed to explore freely for 5 min. The number of grid crossings, rearing events, defecation counts, and average movement speed were recorded. To eliminate olfactory cues, the arena was thoroughly cleaned with 10 % ethanol between trials.

### 2.7 Electrophysiological recording

Mice were anesthetized with isoflurane and decapitated. Their brains were rapidly dissected and placed in ice-cold artificial cerebrospinal fluid (aCSF), continuously bubbled with 95 % O_2_/5 % CO_2_. The aCSF contained 119 mM sucrose, 2.5 mM KCl, 2.5 mM CaCl_2_, 26.2 mM NaHCO_3_, 1.0 mM NaH_2_PO_4_, 1.3 mM MgCl_2_, and 11.0 mM glucose. Coronal hippocampal slices (300 μm) were prepared using a vibrating microtome (Leica VT1200, Wetzlar, Germany) and incubated in oxygenated aCSF at 32 °C for h. Subsequently, fEPSPs were recorded using the MED64 multichannel system (Alpha MED Scientific, Osaka, Japan; 8 × 8 array, 50 μm × 50 μm electrodes, 150 μm spacing, 1 mm × 1 mm area). Baseline responses were recorded every 20 s at −10 μA for min. LTP was induced by three trains of theta-burst stimulation (TBS), each consisting of 10 5-Hz bursts of four pulses at 100 Hz, with 40-s intervals between trains.

### 2.8 Transmission electron microscopy (TEM)

TEM was performed following established protocols with minor modifications [24]. Briefly, dissected cortical and hippocampal tissues were fixed in 2.5 % glutaraldehyde at 4 °C for h, followed by post-fixation in 1 % osmium tetroxide at 4 °C for another h. After thoroughly rinsing with distilled water, the samples were dehydrated through a graded ethanol series and subsequently embedded in epoxy resin. Ultrathin sections were obtained using a Leica ultramicrotome equipped with a DiATOME diamond knife, stained with 2 % uranyl acetate and 2 % lead citrate, and imaged using a Tecnai G2 Spirit transmission electron microscope (FEI Company, Hillsboro, OR, USA).

### 2.9 Western blot

Protein concentrations were determined using a BCA Protein Assay Kit (Beyotime, Shanghai, China). Equal amounts of protein samples were separated by SDS-PAGE (Bio-Rad, Hercules, CA, USA) and transferred onto 0.45 μm PVDF membranes (Millipore, Burlington, MA, USA) via wet transfer (Tanon, Beijing, China). Membranes were blocked in 5 % non-fat milk diluted in 1 × TBST and incubated with primary antibodies (as shown in Table S2) overnight at 4 °C. After three washes with 1 × TBST, membranes were incubated with HRP-conjugated secondary antibodies (CST, Boston, USA) at 37 °C for 1 h, followed by three additional washes. Protein bands were visualized using an enhanced chemiluminescence detection system (Tanon, Beijing, China) and quantified using the ImageJ software.

### 2.10 RNA extraction and quantitative real-time PCR (qPCR)

Total RNA was extracted from mouse cortical or hippocampal tissues using the RNAFast200 kit (Fastagen, Shanghai, China), and complementary DNA (cDNA) was synthesized using the StarScript III All-in-One RT Mix with gDNA Remover (Genstar, Guangzhou, China). qPCR was performed using 2 × RealStar Fast SYBR qPCR Mix (Genstar, Guangzhou, China) on a CFX Connect Real-Time PCR Detection System (Bio-Rad). Relative gene expression levels were normalized to the reference genes *Actin* or *Gapdh*. Primer sequences are listed in Table S3.

### 2.11 Acyl-biotin exchange (ABE)

ABE was performed as previously described [25]. Briefly, approximately 1 mg of total protein was subjected to chloroform–methanol (CM) precipitation, followed by overnight blocking of free thiols with 50 mM N-ethylmaleimide (NEM) at 4 °C. After CM precipitation and multiple methanol washes to remove excess NEM, proteins were resuspended in HPDP-biotin-containing buffer and divided into hydroxylamine-treated (HA^+^) and untreated (HA^−^) groups. The samples were incubated for 2 h at room temperature, followed by CM precipitation and resuspension. The supernatant was incubated with streptavidin–agarose beads for 2 h at room temperature. Beads were thoroughly washed and biotinylated proteins were eluted in sodium dodecyl sulfate (SDS) loading buffer by boiling at 100 °C for 8 min. For mass spectrometry, proteins were eluted in MS-compatible buffer, subjected to another round of CM precipitation, and resuspended in 8 M urea buffer. Samples were stored at −80 °C until further analysis.

### 2.12 Co-immunoprecipitation (Co-IP)

The magnetic beads were gently resuspended and washed thrice with IP lysis buffer. Antibodies were diluted to a final concentration of 5–50 μg/mL in IP lysis buffer and incubated with 400 μL of beads for 1 h at room temperature on a rotator. After three washes, 400 μL of pre-quantified protein lysate from mouse tissue or cultured cells was added and incubated either for h at room temperature or overnight at 4 °C. The beads were then washed thrice, and bound proteins were eluted with 50 μL of 1 × SDS loading buffer by heating at 95 °C for 5 min. After cooling, samples were placed on a magnetic stand and the supernatant was collected for SDS-PAGE and subsequent Western blot analysis.

### 2.13 Immunofluorescence and image analysis

Glass coverslips were pretreated with ethanol, washed with DMEM, and UV-irradiated before cell seeding. After 24 or 48 h, cells were fixed in 4 % paraformaldehyde for 15 min. For tissue analysis, mice were anesthetized (80 mg/kg pentobarbital sodium, i.p.), perfused with PBS, and brains were post-fixed in 4 % paraformaldehyde for 24 h at 4 °C. Following sucrose dehydration and OCT embedding (Sakura, California, USA), coronal sections (10 μm) were prepared. Samples were permeabilized, blocked, and incubated with primary antibodies (as shown in Table S2) overnight at 4 °C. After PBS washes, fluorescent secondary antibodies (as shown in Table S2) were applied for 1 h at 37 °C in the dark. Nuclei were counterstained with 4′,6-diamidino-2-phenylindole (DAPI), and samples were mounted. Images were acquired using a ZEISS LSM 880 confocal microscope (ZEISS, Oberkochen, Germany) or structured illumination microscope (SIM). Colocalization and 3D reconstruction were performed using the Imaris software (v9.0.1, Bitplane).

### 2.14 Proximity ligation assay (PLA)

PLA was performed using the Duolink® In Situ Red Starter Kit (Sigma-Aldrich, St. Louis, MO, USA) with minor modifications. Samples were blocked with Duolink® blocking buffer for 2 h at 37 °C in a humidified chamber, followed by overnight incubation at 4 °C with primary antibodies diluted in antibody diluent. After two washes with 1 × Wash Buffer A, samples were incubated with PLUS and MINUS PLA probes for 2 h at 37 °C. Subsequently, slides were washed and incubated with ligation buffer containing ligase for 1h at 37 °C, followed by amplification with polymerase-containing buffer for 2h at 37 °C. After final washes with 1 × and 0.01 × Wash Buffer B, nuclei were stained with DAPI, and slides were mounted using Duolink® mounting medium. SIM imaging was performed 15 min after mounting.

### 2.15 ER fractionation

ER fractionation was performed using a commercial extraction kit according to the manufacturer's protocol (EX2690; Solarbio, Beijing, China). Fresh brain tissue or cultured cells were rinsed with PBS, minced, and homogenized in 500 μL of ice-cold Reagent A, followed by 10 min of incubation on ice. The homogenate was centrifuged at 1000×*g* for 5 min at 4 °C to remove cell debris. The supernatant was then centrifuged at 11 000×*g* for 10 min and at 16 000×*g* for another 10 min at 4 °C. The resulting supernatant was mixed with 125 μL of Reagent B and incubated overnight at 4 °C. The ER-enriched fraction was collected by centrifugation at 15 000×*g* for 45 min, lysed in RIPA buffer, and stored at −20 °C for subsequent analysis.

## 3 Results

### 3.1 Cognitive and synaptic impairments precede synapse loss in SELENOK-knockout mice

**Fig. 1:**
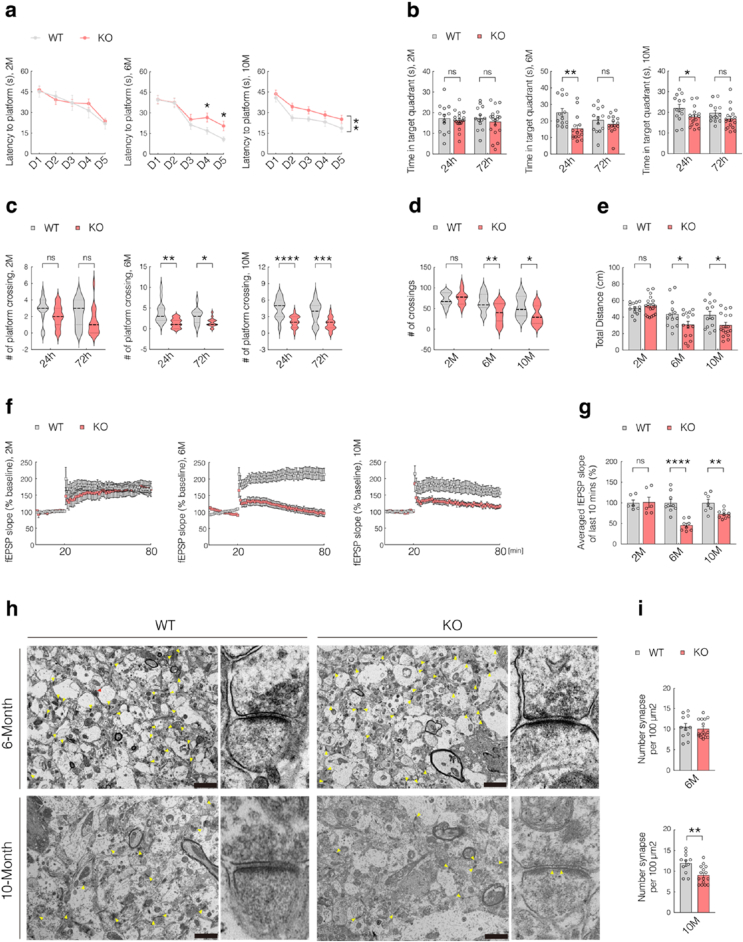
Cognitive and synaptic plasticity deficits in knockout mice at 6 months of age. **(a**–**c)** Morris water maze test evaluating spatial learning and memory in wild-type (WT) and knockout (KO) mice at 2, 6, and 10 months of age. (**a**) Escape latency; (**b**) time spent in the target quadrant during the 24-h and 72-h probe trials; (**c**) number of platform crossings during the 24-h and 72-h probe trials. (n = 13–19; sex-balanced) **(d**–**e)** Open-field test measuring spontaneous locomotor activity. (**d**) Grid crossings; (**e**) Total travel distance. (n = 13–19; sex-balanced) **(f**–**g)** Long-term potentiation (LTP) in the hippocampal Schaffer collateral pathway recorded using an MED64 multi-electrode array system. (**f**) LTP recordings over 80 min; (**g**) average fEPSP amplitude during the final 10 min after high-frequency stimulation. (n = 6–9; sex-balanced). **(h**–**i)** Transmission electron microscopy (TEM) analysis of synapse number and structure. (**h**) Representative TEM images of synapses and (**i**) quantification of synapse number. (Scale bar = 2 μm; n = 11–15; four mice per group; sex-balanced) Data are presented as mean ± SEM. (**a**) Two-way analysis of variance (ANOVA) with Bonferroni post-hoc analysis. Other data were analyzed with Student's t-test. ∗*p* < 0.05; ∗∗*p* < 0.01; ∗∗∗*p* < 0.001; ∗∗∗∗*p* < 0.0001.

Our previous study showed that 7-month-old SELENOK-knockout (SELENOK^−/−^, KO) mice exhibit significant cognitive deficits [7]. To determine the temporal onset of cognitive and synaptic impairments, we evaluated spatial learning and memory in wild-type (WT) and KO mice at 2, 6, and 10 months of age, using the Morris water maze test. Compared to WT controls, KO mice showed significantly prolonged escape latencies at 6 and 10 months, whereas no difference was observed at 2 months (Fig. 1a). In the subsequent 24-h probe trial, 6- and 10-month-old KO mice spent significantly less time in the target quadrant and had fewer platform crossings compared to WT mice (Fig. 1b and c). At 72 h, KO mice continued to show a significantly reduced number of platform crossings, although the time spent in the target quadrant was comparable to that of WT mice (Fig. 1b and c). Furthermore, open-field testing revealed impaired spontaneous locomotor activity in KO mice aged 6 and 10 months, indicated by fewer grid crossings and reduced total travel distance, with no significant differences observed at 2 months (Fig. 1d and e). These findings suggest that measurable cognitive and behavioral impairments in KO mice emerge as early as six months of age.

Given the close association between synaptic plasticity and cognitive function, we evaluated neuronal plasticity by recording long-term potentiation (LTP) in the hippocampal Schaffer collateral pathway using the MED64 multi-electrode array system (Alpha Med Sciences, Kadoma, Japan). High-frequency stimulation effectively induced LTP at 2, 6, and 10 months of age, as indicated by the increased field excitatory postsynaptic potential (fEPSP) amplitudes. However, LTP induction was significantly impaired in 6- and 10-month-old KO mice compared to WT controls, as shown by a marked reduction in the average fEPSP amplitude during the final 10 min of recording (Fig. 1f and g). In contrast, 2-month-old KO mice exhibited no significant differences in LTP magnitude or fEPSP amplitude compared to WT mice (Fig. 1f and g), indicating that synaptic plasticity deficits in KO mice emerge by 6 months of age.

Since synaptic structure and density support functional plasticity, ultrastructural changes were examined using transmission electron microscopy (TEM). At 10 months of age, KO mice exhibited widened synaptic clefts, reduced postsynaptic density (PSD) thickness, and significantly decreased synapse numbers compared to WT controls (Fig. 1h and i). However, no apparent structural abnormalities or synapse loss were observed in 6-month-old KO mice (Fig. 1h and i). Collectively, these findings indicate that cognitive decline and synaptic plasticity impairments precede overt synaptic loss in KO mice.

### 3.2 GluA2 as a potential SELENOK target in synaptic plasticity regulation

**Fig. 2:**
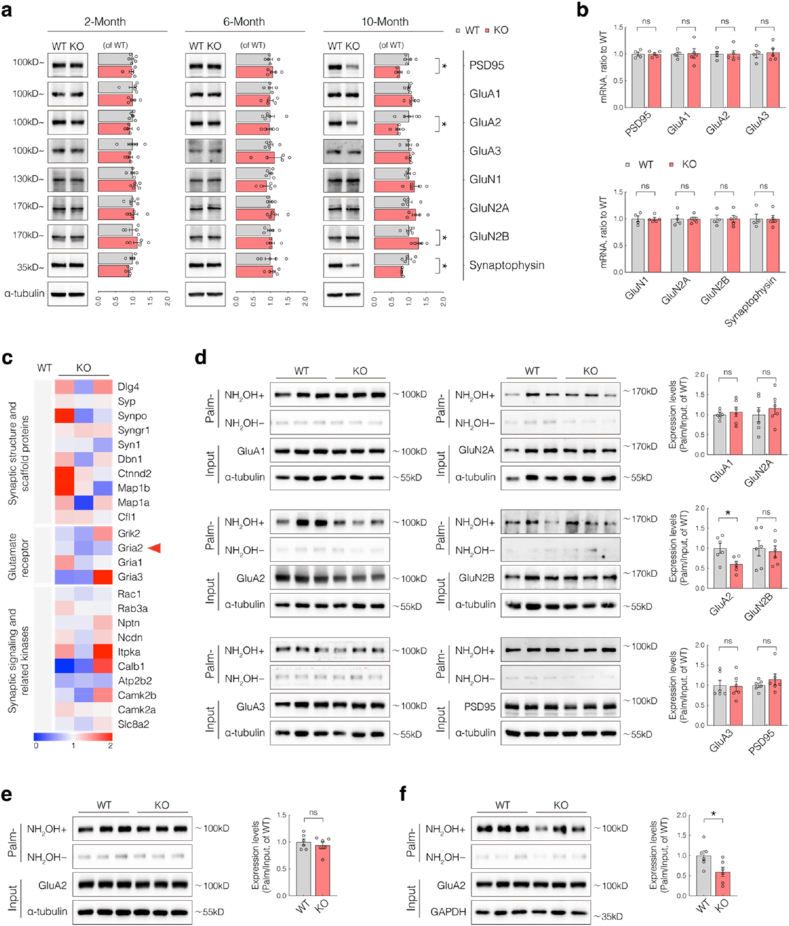
SELENOK Modulates Synaptic Plasticity through GluA2 palmitoylation. (**a**) Western blot (WB) analysis of PSD95, synaptophysin, GluA1, GluA2, GluA3, GluN1, GluN2A, and GluN2B in hippocampal lysates from wild-type (WT) and knockout (KO) mice at 2, 6, and 10 months (n = 5; 3 males and 2 females). (**b**) qPCR analysis of synapse-related gene expression in the hippocampus of 10-month-old WT and KO mice (n = 5; 3 males and 2 females). (**c**) Heatmap of differentially palmitoylated proteins related to synaptic plasticity in hippocampi of 10-month-old WT and KO mice (normalized to WT) (n = 3; 2 males and 1 female). (**d**) Acyl-biotin exchange (ABE) followed by western blotting (ABE-WB) analysis of total and palmitoylated forms of indicated synaptic proteins in 10-month-old WT and KO hippocampi (n = 6; sex-balanced). (**e-f**) ABE-WB analysis of total protein and palmitoylation levels of GluA2 in hippocampi of 2-month-old (e) and 6-month-old (f) WT and KO mice (n = 6; sex-balanced). Data are presented as mean ± SEM and analyzed using Student's t-test. ∗*p* < 0.05.

To elucidate the molecular mechanisms by which SELENOK regulates synaptic plasticity, we analyzed the expression of synapse-associated proteins, including structural proteins (PSD95, synaptophysin) and plasticity-related receptor subunits (GluA1, GluA2, GluA3, GluN1, GluN2A, GluN2B), in hippocampal tissues from WT and KO mice aged 2, 6, and 10 months. In 10-month-old KO mice, we observed significant reductions in PSD95, synaptophysin, and GluA2 levels, along with a marked increase in GluN2B levels, whereas the other receptor subunits showed no significant changes (Fig. 2a). Importantly, none of these proteins showed significant changes in their expression at 2 or 6 months (Fig. 2a). The delayed onset of synaptic protein changes relative to the appearance of cognitive and synaptic deficits suggests that these phenotypes are unlikely to be directly caused by synaptic protein dysregulation. However, quantitative real-time polymerase chain reaction (qPCR) analysis revealed no significant differences in mRNA levels of these proteins at 10 months (Fig. 2b), indicating that the observed protein-level changes may result from altered translation, post-translational modifications (PTMs), or protein degradation. These findings point to GluA2 as a potential SELENOK target involved in regulating synaptic plasticity.

SELENOK has been implicated in the regulation of protein palmitoylation, a lipid-based PTM that modulates protein stability, membrane targeting, and trafficking. Notably, approximately 10 % of all proteins—and over 40 % of synaptic proteins—are palmitoylated [26]. To identify palmitoylation-dependent targets of SELENOK, acyl-biotin exchange (ABE) coupled with mass spectrometry was performed on hippocampal tissue from 10-month-old KO mice. Among the synaptic plasticity–related palmitoylated proteins, GluA2 and ATP2B2 consistently showed reduced palmitoylation across three biological replicates (Fig. 2c), suggesting SELENOK-dependent regulation of palmitoylation. ABE followed by western blotting further confirmed that GluA2 palmitoylation was markedly reduced in KO hippocampi, whereas no significant changes were observed in the other candidates (Fig. 2d). These findings suggest that SELENOK modulates synaptic plasticity by regulating the palmitoylation of GluA2.

To determine the temporal onset of the dysregulation of GluA2 palmitoylation, hippocampal tissues from 2- and 6-month-old mice were analyzed. While no difference was observed at two months, a significant reduction in GluA2 palmitoylation was detected in 6-month-old KO mice (Fig. 2e and f), coinciding with the emergence of cognitive deficits and impaired synaptic plasticity. These findings identify GluA2 palmitoylation as a potential molecular target underlying SELENOK-mediated regulation of synaptic function.

### 3.3 SELENOK regulates GluA2 palmitoylation through DHHC6

Although SELENOK lacks the canonical Asp-His-His-Cys (DHHC) catalytic motif required for palmitoyltransferase activity, it has been shown to regulate protein palmitoylation through interaction with the palmitoyl acyltransferase DHHC6. Although DHHC6 is localized to the ER—the same subcellular site where GluA2 palmitoylation occurs—it remains unclear whether DHHC6 directly catalyzes GluA2 palmitoylation.

**Fig. 3:**
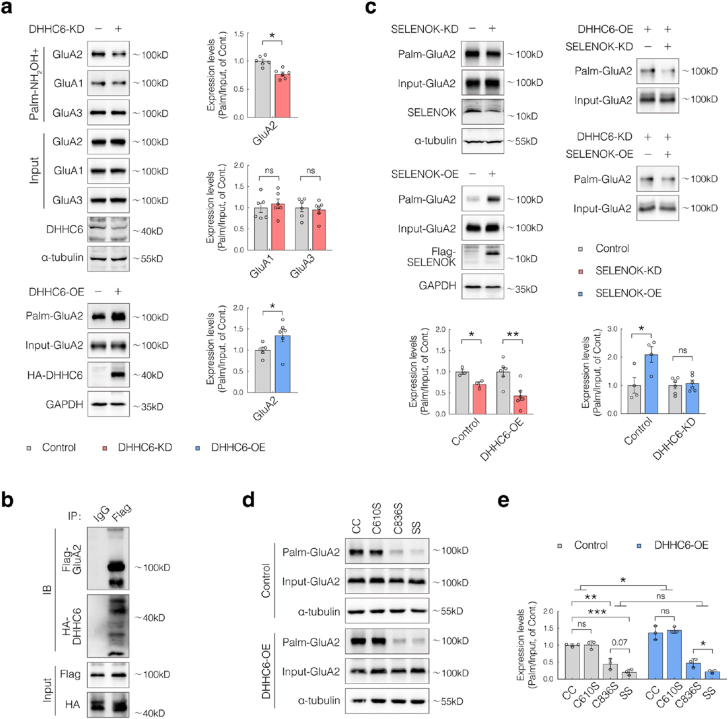
SELENOK facilitates DHHC6-mediated palmitoylation of GluA2 at Cys836. (**a**) HEK293 cells were co-transfected with sh-DHHC6 and Flag-tagged GluA constructs (GluA1, GluA2, or GluA3). Total and palmitoylation protein levels were assessed by acyl-biotin exchange (ABE) followed by western blotting (ABE-WB) (n = 6). (**b**) Co-immunoprecipitation (Co-IP) followed by western blotting (Co-IP/WB) analysis in HEK293 cells co-expressing Flag-GluA2 and HA-DHHC6 to assess protein interaction. (**c**) In HEK293 cells with adenoviral-mediated SELENOK knockdown (SELENOK-KD) or overexpression (SELENOK-OE), Flag-GluA2 was co-transfected with HA-DHHC6, sh-DHHC6, or control vectors. ABE-WB was used to analyze GluA2 palmitoylation (n = 3–5). (d–e) HEK293 cells were transfected with either an empty vector or HA-DHHC6, together with wild-type Flag-GluA2 (CC), single-point mutants (C610S and C836S), or the double mutant (C610S/C836S, referred to as SS). ABE-WB was performed to assess GluA2 palmitoylation (n = 3). Data are presented as mean ± SEM and analyzed using Student's t-test. ∗*p* < 0.05; ∗∗*p* < 0.01; ∗∗∗*p* < 0.001.

To address this, DHHC6 knockdown (DHHC6-KD) HEK293 cells were generated and transfected with GluA1, GluA2, or GluA3 expression constructs. Silencing DHHC6 markedly reduced GluA2 palmitoylation, whereas the palmitoylation levels of GluA1 and GluA3 remained unchanged (Fig. 3a). In contrast, DHHC6 overexpression (DHHC6-OE) significantly increased GluA2 palmitoylation (Fig. 3a). Co-expression of Flag-GluA2 and HA-DHHC6 in HEK293 cells, followed by co-immunoprecipitation, further confirmed a robust interaction between GluA2 and DHHC6 (Fig. 3b). These findings indicate that DHHC6 directly catalyzes GluA2 palmitoylation.

Since SELENOK modulates GluA2 palmitoylation, its regulatory role was validated in vitro. Adenoviral-mediated SELENOK knockdown significantly reduced GluA2 palmitoylation, whereas its overexpression enhanced it (Fig. 3c). Notably, in DHHC6-KD cells, SELENOK overexpression failed to restore GluA2 palmitoylation, suggesting that SELENOK acts through DHHC6 (Fig. 3c). Furthermore, SELENOK knockdown significantly reduced GluA2 palmitoylation even in DHHC6-overexpressing cells (Fig. 3c), indicating the necessity of SELENOK for efficient DHHC6-mediated GluA2 palmitoylation.

GluA2 contains two established palmitoylation sites—Cys610 and Cys836 [27]. To identify the functional site catalyzed by DHHC6, single-point mutants (C610S and C836S) and a double mutant (C610S/C836S, hereafter referred to as SS) were generated and co-expressed with DHHC6 in HEK293 cells. Compared with wild-type GluA2 (CC), both the C836S and SS mutants showed significantly reduced palmitoylation levels, with the SS mutant exhibiting a slightly greater reduction, whereas the C610S mutant showed no significant difference (Fig. 3d and e). Furthermore, DHHC6 overexpression significantly enhanced palmitoylation in the CC and C610S constructs but had no effect on the C836S or SS mutants (Fig. 3d and e). These findings identify Cys836 as the primary site of DHHC6-catalyzed GluA2 palmitoylation.

### 3.4 SELENOK is involved in AMPA receptor (AMPAR) complex assembly

**Fig. 4:**
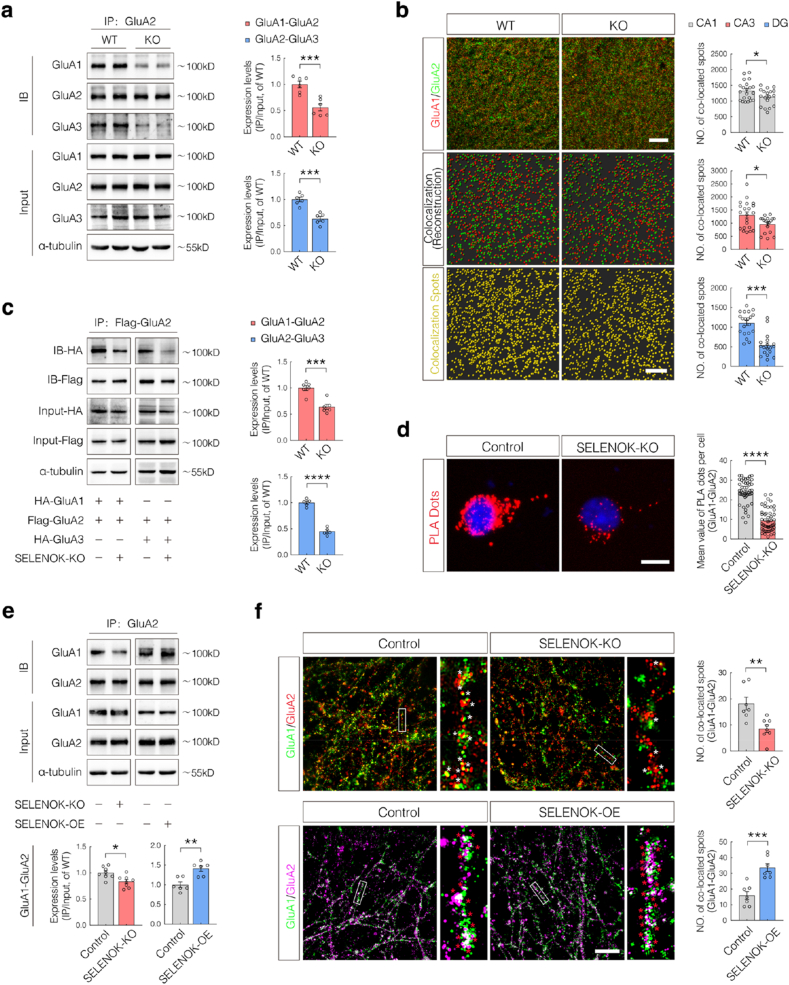
SELENOK is involved in AMPAR complex assembly. **(a)** Co-immunoprecipitation (Co-IP)/western blotting (WB) analysis of GluA1–GluA2 and GluA2–GluA3 interactions in the hippocampi of 6-month-old WT and KO mice (n = 6; sex-balanced). **(b)** Immunofluorescence (IF) and structured illumination microscopy (SIM) imaging to visualize the co-localization of GluA1 (red) and GluA2 (green) in hippocampal sections, with colocalized spots appearing yellow (scale bar = 8 μm). Quantification was performed using the Imaris 3D reconstruction (scale bar = 10 μm; n = 3; 7 non-overlapping, equal-area regions per mouse; 2 males and 1 female per group) **(c)** Co-IP/WB analysis of Flag-GluA2 interactions with HA-GluA1 or HA-GluA3 in SELENOK-KO N2a cells (n = 5–6). **(d)** Proximity ligation assay (PLA) quantifying GluA1–GluA2 interactions in SELENOK-KO N2a cells, with PLA signals shown in red (scale bar = 10 μm; n = 40). **(e)** Co-IP/WB analysis of GluA1–GluA2 interactions in primary neurons with SELENOK knockdown (SELENOK-KO) or overexpression (SELENOK-OE) (n = 6). **(f)** IF and SIM imaging of GluA1–GluA2 colocalization in primary neurons under SELENOK-KO or SELENOK-OE conditions (scale bar = 10 μm; n = 7 fields from 3 slices) Data are presented as mean ± SEM and analyzed using Student's t-test. ∗*p* < 0.05; ∗∗*p* < 0.01; ∗∗∗*p* < 0.001; ∗∗∗∗*p* < 0.0001.

Mature AMPAR complexes primarily exist as GluA1-GluA2 and GluA2-GluA3 heteromers, with GluA2 playing a critical role in their assembly. Since AMPAR assembly occurs in the ER, and GluA2 is uniquely palmitoylated within this compartment [28], we hypothesized SELENOK may mediate AMPAR complex formation through its regulation of GluA2 palmitoylation. Co-immunoprecipitation (Co-IP) analyses revealed significantly reduced GluA1–GluA2 and GluA2–GluA3 interactions in hippocampal tissues from 6-month-old KO mice compared to WT controls (Fig. 4a). Consistently, super-resolution structured illumination microscopy (SIM) and 3D reconstruction showed markedly decreased GluA1–GluA2 colocalization across hippocampal subregions (CA1, CA3, and DG) in KO mice (Fig. 4b).

To strengthen these findings, SELENOK-knockout (SELENOK-KO) Neuro-2a (N2a) cells generated via CRISPR-Cas9 exhibited substantially weakened GluA1–GluA2 and GluA2–GluA3 interactions, as determined by the co-expression of Flag-GluA2 with HA-GluA1 or HA-GluA3 (Fig. 4c). These results were corroborated by a proximity ligation assay (PLA), which showed significantly reduced PLA signals in SELENOK-KO cells (Fig. 4d). Similar patterns were observed in primary cultured neurons: SELENOK deletion reduced GluA1–GluA2 interactions, whereas SELENOK overexpression significantly enhanced them (Fig. 4e). Immunofluorescence analysis further revealed decreased GluA1–GluA2 colocalization in SELENOK-KO neurons, which was reversed by SELENOK overexpression (Fig. 4f). Collectively, these in vivo and in vitro findings highlight a critical role for SELENOK in regulating AMPAR complex assembly.

### 3.5 SELENOK promotes AMPAR assembly through palmitoylation-dependent regulation of the ER GluA2 pool

The above findings indicate that SELENOK plays a role in regulating AMPAR complex assembly. However, whether this effect is mediated specifically through its regulation of GluA2 palmitoylation and whether GluA2 palmitoylation within the ER directly facilitates AMPAR assembly remains unclear. Previous studies have shown that non-palmitoylated GluA2 is preferentially trafficked to lysosomes for degradation [18], suggesting that ER-localized GluA2 palmitoylation is essential for proper complex formation. Given the established role of palmitoylation in promoting protein stability, it has been proposed that palmitoylated GluA2 is stabilized and retained within the ER, thereby supporting the formation of an ER-localized GluA2 pool that may be critical for efficient AMPAR complex assembly.

**Fig. 5:**
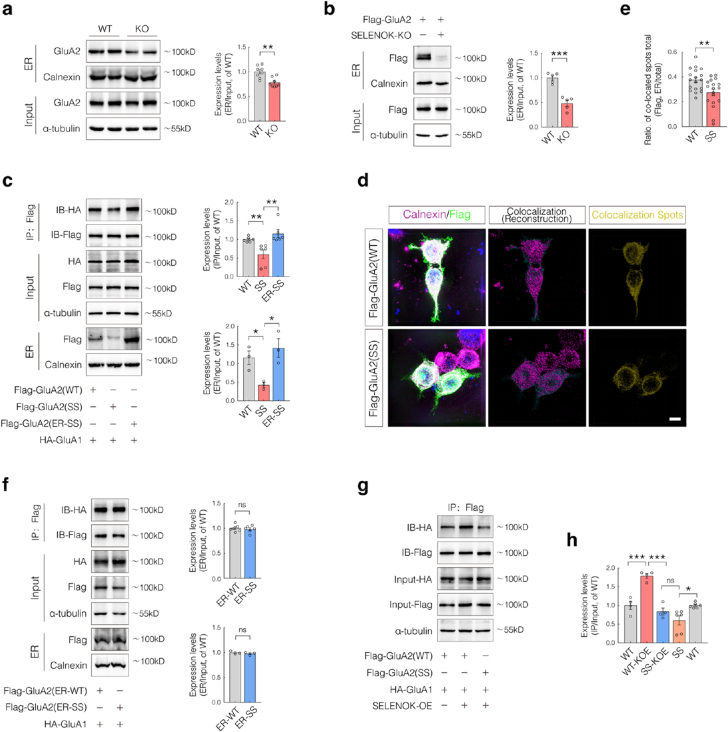
SELENOK regulates ER-localized GluA2 via palmitoylation to regulate AMPAR assembly. (**a**) ER fractions were isolated from the hippocampi of 6-month-old wild-type (WT) and knockout (KO) mice using an ER isolation kit, followed by WB analysis of ER-localized GluA2 (n = 8; sex-balanced). (**b**) SELENOK-KO N2a cells exogenously expressing Flag-GluA2 were subjected to ER fractionation, and ER-localized GluA2 levels were analyzed by WB (n = 5). (**c**) N2a cells were co-transfected with HA-GluA1 and Flag-GluA2 (WT, SS, or ER-SS). GluA1–GluA2 interactions were assessed by co-immunoprecipitation and western blotting (Co-IP/WB), and ER-localized GluA2 levels analyzed by WB after ER fractionation (n = 6 or 3). **(d**–**e)** Immunofluorescence **(**IF) and structured illumination microscopy (SIM) imaging of N2a cells expressing Flag-GluA2-WT or Flag-GluA2-SS, showing colocalization with the ER marker calnexin (colocalized spots are shown in yellow; scale bar = 8 μm; n = 18). (**f**) N2a cells were co-transfected with HA-GluA1 and ER-targeted Flag-GluA2 (ER-WT or ER-SS), followed by Co-IP/WB to assess GluA1–GluA2 interactions and WB analysis of ER-localized GluA2 levels after ER fractionation (n = 6 or 3). (**g–h**) N2a cells were co-transfected with HA-GluA1 and either WT or SS Flag-GluA2 along with a SELENOK overexpression plasmid. GluA1–GluA2 interactions were analyzed by CO-IP/WB. (**h**) Quantification of interactions in WT and SS groups from SELENOK-overexpressing cells (**g**) and corresponding controls from (**c**). (n = 4). Data are presented as mean ± SEM and analyzed using Student's t-test. ∗*p* < 0.05; ∗∗*p* < 0.01; ∗∗∗*p* < 0.001.

To test this hypothesis, ER fractions were isolated from the hippocampal tissues of 6-month-old mice. A significant reduction in ER-localized GluA2 was observed in KO mice (Fig. 5a). Consistent results were obtained in N2a cell models, where SELENOK-KO led to a marked decrease in ER-retained Flag-GluA2 (Fig. 5b), indicating that SELENOK is essential for maintaining GluA2 retention within the ER. To assess whether palmitoylation of GluA2 is essential for its ER localization and subsequent AMPAR assembly, N2a cells were transfected with plasmids expressing either WT or SS GluA2, along with HA-GluA1. Compared to WT, the SS mutant exhibited reduced ER accumulation and a weakened interaction with GluA1 (Fig. 5c and d), suggesting that the loss of GluA2 palmitoylation impairs both ER retention and AMPAR complex formation.

To further assess whether restoring ER localization can compensate for the loss of palmitoylation, an ER-retention sequence (KDEL) was fused to the palmitoylation-deficient GluA2 mutant ER-SS. Notably, ER-SS restored both the levels of ER-localized GluA2 and its interaction with GluA1, with the latter slightly surpassing that of the WT group (Fig. 5c). These findings indicate that stabilizing GluA2 retention within the ER—independent of palmitoylation—is sufficient to promote AMPAR complex assembly, highlighting a mechanistic link between GluA2 palmitoylation, ER pool formation, and AMPAR assembly.

Although the palmitoylation sites of GluA2 are located outside the known subunit interaction domains [29], this modification may still indirectly influence GluA2 conformation to affect complex formation. To validate this, KDEL-tagged WT and SS GluA2 constructs (referred to as ER-WT and ER-SS, respectively) were co-transfected with HA-GluA1 in N2a cells. No significant differences were observed between the two groups in either GluA1–GluA2 interaction or ER-localized GluA2 levels (Fig. 5f), indicating that palmitoylation promotes AMPAR assembly primarily by increasing ER-localized GluA2 levels, rather than by altering subunit-binding compatibility. To further confirm whether SELENOK acts through this mechanism, N2a cells were co-transfected with WT or SS GluA2, HA-GluA1, and a SELENOK overexpression plasmid. SELENOK overexpression markedly enhanced GluA1–GluA2 interactions in the WT group but had no effect in the SS group (Fig. 5g and h). These findings establish GluA2 palmitoylation as a key mediator of SELENOK-dependent AMPAR assembly by linking the ER-resident GluA2 pool to synaptic function.

### 3.6 Both GluA2 palmitoylation and AMPAR assembly are impaired in AD

Our previous studies have shown that SELENOK expression is significantly reduced in the brains of both patients with AD and mouse models of AD [4,8]. Moreover, SELENOK-KO mice exhibit neuropathological phenotypes resembling those of AD, including disrupted intracellular Ca^2+^ homeostasis and synaptic deficits [4]. These findings led to the hypothesis that the downregulation of SELENOK in AD contributes to reduced GluA2 palmitoylation and impaired AMPAR assembly.

**Fig. 6:**
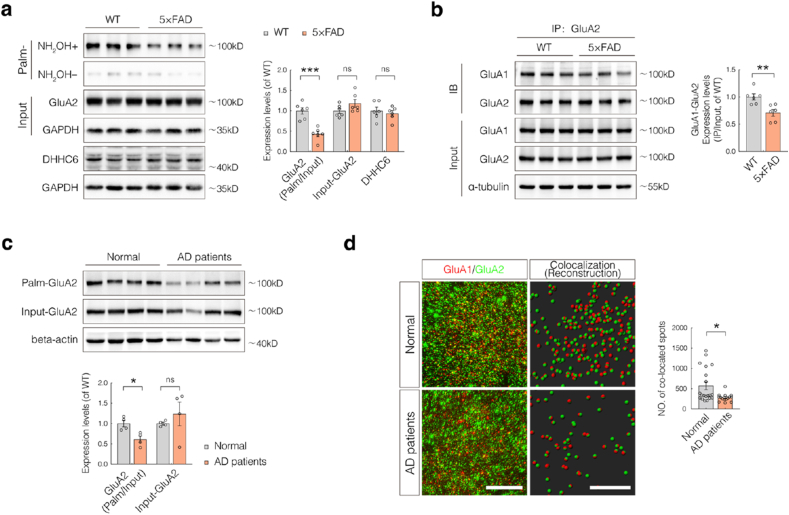
The AD Brain exhibits reduced GluA2 palmitoylation and impaired AMPAR assembly. (**a**) Acyl-biotin exchange-western blotting (ABE-WB) analysis of GluA2 palmitoylation and total protein levels of GluA2 and DHHC6 in the hippocampi from 6-month-old wild-type (WT) and 5 × FAD transgenic mice (n = 6; sex-balanced). (**b**) Co-immunoprecipitation (Co-IP)/WB analysis of GluA1–GluA2 interactions in the hippocampi from 6-month-old WT and 5 × FAD transgenic mice (n = 6; sex-balanced). (**c**) ABE-WB analysis of GluA2 palmitoylation and total protein levels in postmortem cortical tissues from normal controls and patients with AD. (n = 4) **(d)** Immunofluorescence (IF) and structured illumination microscopy (SIM) imaging to assess GluA1–GluA2 colocalization in hippocampal sections. GluA1 (red), GluA2 (green), and co-localized signals (yellow) (scale bar = 8 μm). Quantification was performed using Imaris 3D reconstruction (scale bar = 10 μm; n = 4 controls, 3 patients with AD; 5 non-overlapping, equal-area regions per section). Data are presented as mean ± SEM and analyzed using Student's t-test. ∗*p* < 0.05; ∗∗*p* < 0.01; ∗∗∗*p* < 0.001.

Compared to wild-type mice, 6-month-old 5 × FAD transgenic mice—a widely used model of AD—exhibited a significant reduction in GluA2 palmitoylation levels in the hippocampus, whereas the total protein levels of GluA2 and its primary palmitoyltransferase DHHC6 remained unaltered (Fig. 6a). Furthermore, Co-IP analysis revealed a substantial decrease in the GluA1–GluA2 interaction (Fig. 6b), indicating compromised AMPAR assembly under AD pathological conditions.

Consistent results were observed in postmortem cortical tissues from patients with AD, which exhibited significantly reduced GluA2 palmitoylation compared to cognitively normal controls, despite comparable total GluA2 protein levels (Fig. 6c). Additionally, immunofluorescence staining and SIM imaging of hippocampal sections showed a marked decrease in GluA1–GluA2 colocalization in patients with AD (Fig. 6d). Collectively, these findings provide converging evidence from both human and mouse models that AD is associated with reduced GluA2 palmitoylation and impaired AMPAR assembly.

### 3.7 Neuronal SELENOK overexpression restores cognition and AMPAR assembly in 5 × FAD mice

**Fig. 7:**
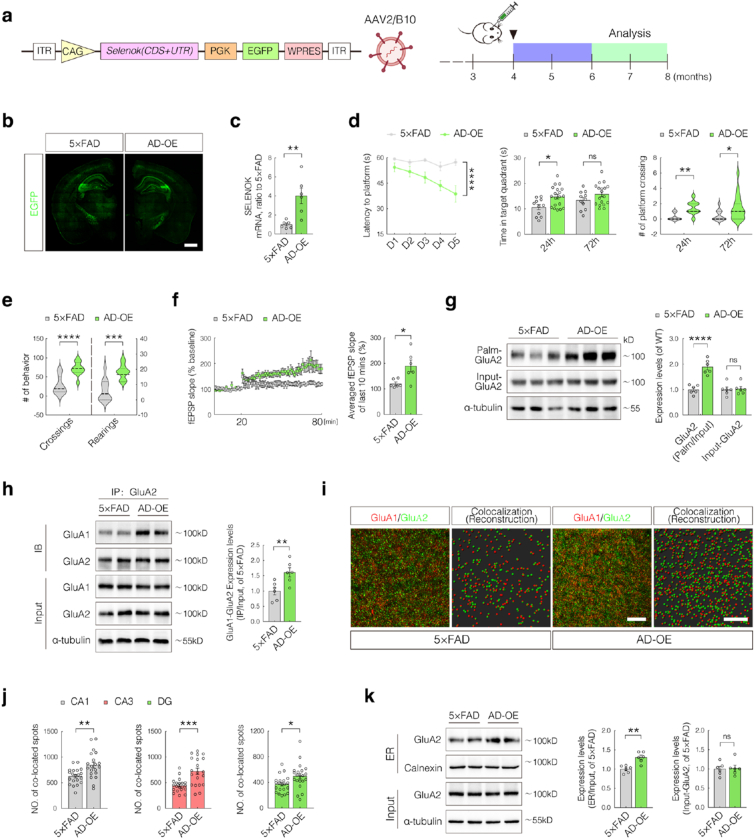
Neuronal SELENOK overexpression restores AMPAR function and cognitive impairment in 5 × FAD transgenic mice. (**a**) Schematic of the AAV2/B10 vector encoding SELENOK and experimental timeline for virus injection and behavioral testing. (**b**) Representative fluorescence image of enhanced green fluorescent protein (EGFP)-labeled adeno-associated virus (AAV) expression in brain tissue (scale bar = 1000 μm). (**c**) Quantitative real-time PCR (qPCR) analysis of SELENOK expression in the hippocampi of AD and AD-OE mice (n = 6; sex-balanced). (**d**) Morris water maze performance in 5 × FAD and AD-OE mice. **Left**: Escape latency; **Middle**: Time spent in the target quadrant during 24 h and 72 h post-training; **Right**: Number of platform crossings during 24 h and 72 h probe trials (n = 11 or 19; sex-balanced). (**e**) Open-field test evaluating spontaneous locomotor activity. **Left**: Grid crossings; **Right**: Rearing events (n = 11 or 19; sex-balanced). (**f**) Long-term potentiation (LTP) recordings in the hippocampal Schaffer pathway using the MED64 system. **Left**: Field excitatory postsynaptic potential (fEPSP) amplitude over 80 min; **Right**: Average fEPSP amplitude during the final 10 min after high-frequency stimulation (n = 6; sex-balanced). (**g**) Acyl-biotin exchange-western blotting (ABE-WB) analysis of GluA2 palmitoylation and total protein levels in the hippocampus (n = 6; sex-balanced). (**h**) Co-IP/WB analysis of GluA1–GluA2 interactions in hippocampal lysates (n = 6; sex-balanced). (**i**) Immunofluorescence (IF) and structured illumination microscopy (SIM) imaging of GluA1–GluA2 colocalization in the CA1, CA3, and DG regions (scale bar = 8 μm). (**j**) Quantification of colocalization using Imaris 3D reconstruction. GluA1 (red), GluA2 (green), colocalized (yellow) (scale bar = 10 μm; n = 4; 5 non-overlapping, equal-area regions per section). (**k**) WB analysis of ER-localized GluA2 in hippocampal samples following ER fractionation (n = 6; sex-balanced). Data are presented as mean ± SEM. Data from (**d, left**) were analyzed using Two-way ANOVA and Bonferroni post-hoc analyses were used for data from panel (d, left). Student's t-test was used for all other comparisons. ∗*p* < 0.05; ∗∗*p* < 0.01; ∗∗∗*p* < 0.001; ∗∗∗∗*p* < 0.0001.

To determine whether enhancing SELENOK-mediated GluA2 palmitoylation and AMPAR assembly could alleviate cognitive and synaptic deficits in AD, we used a neuron-specific adeno-associated virus (AAV2/B10) to overexpress SELENOK (AD-OE) in 4-month-old 5 × FAD mice through retro-orbital injection, selectively targeting hippocampal neurons (Fig. 7a). Fluorescence imaging confirmed successful viral transduction, showing robust expression in the hippocampus (Fig. 7b). Additionally, qPCR verified a significant upregulation of SELENOK expression in the hippocampus (Fig. 7c), validating the effectiveness of the viral delivery.

Two months post-injection, spatial learning and memory were evaluated using the Morris water maze test. AD-OE mice showed significantly reduced escape latencies compared to AD controls (Fig. 7d). In the 24-h probe trial, both the time spent in the target quadrant and the number of platform crossings were significantly increased (Fig. 7d). At 72 h, the number of platform crossings remained significantly elevated, while the time spent in the target quadrant showed no significant difference (Fig. 7d). Open-field testing further revealed enhanced exploratory behavior in AD-OE mice, with more grid crossings and rearing events (Fig. 7e), indicating partial restoration of spontaneous locomotor activity. Furthermore, electrophysiological recordings showed significantly enhanced synaptic plasticity in AD-OE mice, characterized by a robust increase in LTP and a significantly higher mean fEPSP amplitude during the final 10-min recording window (Fig. 7f).

Mechanistically, SELENOK overexpression significantly increased GluA2 palmitoylation in the hippocampus (Fig. 7g). Co-IP analysis revealed enhanced GluA1–GluA2 interactions (Fig. 7h), whereas immunofluorescence combined with SIM and 3D reconstruction further confirmed enhanced colocalization of the two subunits across the CA1, CA3, and DG regions, indicating improved AMPAR assembly (Fig. 7i and j). Consistent with this mechanism, ER fractionation and immunoblotting showed a significant increase in ER-localized GluA2 in AD-OE mice (Fig. 7k), indicating that SELENOK promotes GluA2 stabilization and retention within the ER, facilitating the formation of an ER-resident GluA2 pool critical for efficient AMPAR assembly. Collectively, these findings indicate that the neuronal overexpression of SELENOK enhances GluA2 palmitoylation and ER localization, promotes AMPAR complex formation, and alleviates synaptic and cognitive deficits in AD mice.

## 4 Discussion

Se and selenoproteins are essential for maintaining cognitive and synaptic function; however, the underlying molecular mechanisms remain unclear. In this study, GluA2 palmitoylation was identified as a key downstream target of SELENOK in the regulation of synaptic plasticity. Mechanistically, SELENOK promotes the formation of an ER-resident GluA2 pool through palmitoylation, thereby enhancing AMPAR assembly and supporting synaptic transmission. Notably, AD-related findings supported this mechanism, as neuronal SELENOK overexpression effectively restored synaptic plasticity and improved cognitive function in AD mice.

Integrated analyses of behavioral performance, synaptic physiology, and receptor expression revealed a regulatory mechanism involving PTMs. While palmitoyl-proteomics identified GluA2 as a direct downstream substrate of SELENOK, a key mechanistic insight from this study is the link between palmitoylation-dependent ER retention of GluA2 and AMPAR assembly. Palmitoylation is a reversible lipid modification that regulates protein stability, localization, and interactions. The unchanged total GluA2 levels in SELENOK-deficient mice suggest that SELENOK regulates GluA2 trafficking or subunit assembly, rather than its expression. Previous studies have shown that ER-to-Golgi trafficking of AMPAR primarily depends on the N-terminal domains of GluA1 and GluA3 [30], suggesting a distinct role for GluA2 in complex assembly. Notably, GluA2 is the only AMPAR subunit known to undergo palmitoylation within the ER, where AMPARs are assembled. This spatial and temporal alignment supports the conclusion that SELENOK promotes AMPAR assembly by enhancing the ER-localized palmitoylation of GluA2, as demonstrated in this study.

While individual AMPAR subunits function independently as non-selective cation channels (e.g., Na^+^ influx) [31], GluA2-containing AMPARs are indispensable for synaptic plasticity because of their edited glutamine-to-arginine (Q/R) site, which regulates Ca^2+^ permeability [32]. While LTP can be initiated without GluA2, its long-term maintenance requires GluA2 to stabilize synaptic transmission and prevent Ca^2+^ toxicity [33]. Thus, GluA2 serves as the core component of AMPARs, predominantly assembling into GluA1–GluA2 and GluA2–GluA3 heteromers. Although the structural determinants of GluA2 interactions with other subunits are well characterized [34], our study uncovers a novel regulatory mechanism affecting AMPAR assembly efficiency: the ER-localized GluA2 pool. This intracellular reservoir ensures a rapid and sufficient GluA2 supply for dynamic AMPAR assembly during LTP maintenance. Although the presence of such a pool was previously hypothesized [18], its experimental validation and functional relevance remain unestablished. Our study provides compelling evidence for the existence of an ER-resident GluA2 pool and elucidates its critical role in facilitating AMPAR assembly and maintaining synaptic plasticity.

**Fig. 8:**
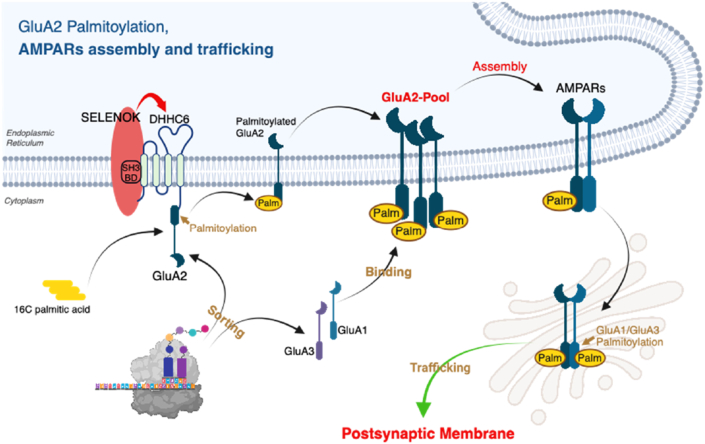
**Proposed mechanism by which SELENOK regulates AMPAR assembly through GluA2 palmitoylation.** SELENOK interacts with the palmitoyl acyltransferase DHHC6 in the ER, promoting DHHC6-mediated palmitoylation of GluA2. Palmitoylated GluA2 is retained in the ER and sorted into a GluA2 pool, which efficiently assembles with GluA1 and GluA3 subunits to form AMPAR complexes. These complexes are subsequently trafficked to the postsynaptic membrane, where they support synaptic transmission and plasticity. SELENOK-dependent GluA2 palmitoylation thus constitutes a key regulatory step in AMPAR assembly, trafficking, and the maintenance of excitatory synaptic function.

Palmitoylation affects over 10 % of gene products, including nearly half of all synaptic proteins and most synaptic receptors [26]. Among the AMPAR subunits, GluA1 and GluA3 are primarily palmitoylated by the Golgi-localized enzyme DHHC3, which facilitates their trafficking and stabilization at the plasma membrane [27]. However, the enzyme mediating GluA2 palmitoylation and the functional significance of its ER-specific modification have remained elusive. In this study, we identified DHHC6 as the key enzyme responsible for GluA2 palmitoylation in the ER and demonstrated that this modification is critical for maintaining an ER-localized GluA2 pool. Mechanistically, our findings delineate a regulatory cascade—“SELENOK–DHHC6–GluA2 palmitoylation–ER GluA2 pool–AMPAR assembly–synaptic plasticity (As shown in Fig. 8).” Although direct evidence linking GluA2 palmitoylation deficits to impaired synaptic plasticity remains limited in this study, the well-established role of GluA2-containing AMPARs in LTP underscores the physiological relevance of this modification.

Additionally, two putative palmitoylation sites have been identified in the C-terminal domain of GluA2: C610 and C836. Our results confirmed palmitoylation at C836, whereas C610 remained unmodified under basal conditions. Notably, palmitoylation at C610 has been reported only in the presence of DHHC3/GODZ or in response to specific stimuli [27]. Whether this site contributes to the regulatory cascade identified in this study, or holds broader physiological relevance, remains to be clarified.

Dysregulated palmitoylation has been implicated in AD pathology. A ZDHHC21 mutation impairs cognition by altering the palmitoylation of amyloid precursor protein (APP), crucial for amyloid-beta (Aβ) production, and FYN kinase, crucial for synaptic signaling and plasticity, leading to Aβ accumulation, tau hyperphosphorylation, and neuronal loss [35]. Similarly, inhibiting beta-site APP cleaving enzyme 1 (BACE1) palmitoylation reduces Aβ production and alleviates cognitive deficits [36]. Our finding that SELENOK expression was markedly reduced in the hippocampus of both patients with AD and model mice suggests that SELENOK-mediated GluA2 palmitoylation may also be impaired in AD. Consistent with this, neuronal overexpression of SELENOK in AD mice alleviated synaptic deficits, implicating GluA2 palmitoylation as a potential mechanism underlying the therapeutic effects of Se in AD.

Interestingly, 7 of the 25 identified selenoproteins—SELENOF, SELENOK, SELENOM, SELENOS, SELENON, SELENOT, and DIO2—are localized to the ER and are highly expressed in the brain [37,38]. In addition to SELENOK, which is linked to palmitoylation, several other ER-resident selenoproteins are involved in distinct PTMs: SELENOT interacts with the oligosaccharyltransferase complex and likely participates in N-glycosylation [39]; SELENOS regulates tau phosphorylation in neurons [40]; and DIO2 is subject to ubiquitination and proteasomal degradation [41]. However, whether these selenoproteins function synergistically or independently in the context of AD remains to be elucidated. Nevertheless, our findings, along with previous evidence, suggest that PTMs mediated by ER-resident selenoproteins could represent a shared mechanism underlying the anti-AD effects of Se, offering a promising direction for future research.

Emerging evidence indicates that redox-dependent hormetic stress activates a cellular “resilience network,” whose core components are vitagenes, which encode cytoprotective proteins such as heat shock proteins, thioredoxin, and heme oxygenase-1 [42]. These adaptive responses precisely regulate protein quality control, mitochondrial bioenergetics, and metabolic homeostasis, thereby effectively counteracting chronic proteotoxicity and neurodegenerative processes. Within this framework, ER-resident selenoproteins, through the regulation of PTMs of proteins, may represent a critical node in the resilience network. This linkage integrates the redox-homeostatic functions of Se and selenoproteins with their distinct molecular regulatory mechanisms, offering a broader perspective for understanding the multifaceted anti-AD actions of Se.

In conclusion, this study provides a comprehensive characterization of SELENOK's role in regulating GluA2 palmitoylation and its implications for synaptic function in AD. We show that SELENOK, through the palmitoyltransferase DHHC6, mediates GluA2 palmitoylation, promoting AMPAR assembly and supporting synaptic plasticity. These findings not only provide new insights into the functional relationship between selenoproteins and synaptic regulation, but also advance our understanding of AD pathogenesis and the mechanisms underlying the therapeutic effects of Se- and Se-containing compounds.

## CRediT authorship contribution statement

**Jiaying Peng:** Project administration, Methodology, Investigation, Data curation. **Zhiyu Cai:** Methodology, Investigation. **Pei Ouyang:** Investigation. **Shujing Lin:** Investigation. **Shurui Zhang:** Investigation. **Danchan Liang:** Investigation. **Ziqi Feng:** Investigation. **Changbin Chen:** Investigation. **Xilin Ye:** Investigation. **Guoli Song:** Writing – review & editing. **Zhonghao Zhang:** Writing – review & editing, Writing – original draft, Project administration, Methodology, Investigation, Funding acquisition, Data curation.

## Declaration of competing interest

The authors declare that they have no known competing financial interests or personal relationships that could have appeared to influence the work reported in this paper.

## Data Availability

Data will be made available on request.
