# Supplementary material for: GluA2 palmitoylation by SELENOK modulates AMPAR assembly and synaptic plasticity in Alzheimer's disease
Source: Redox Biol. 2025 Aug 21;86:103831. doi: 10.1016/j.redox.2025.103831 (PMC12446660; doi:10.1016/j.redox.2025.103831)
Supplement: Multimedia component 1 [file mmc1.docx]

**Table S1 Human demographics.**

| **Diagnosis** | **Sex** | **Age** |
| --- | --- | --- |
| NCI | F | 72 |
| NCI | F | 76 |
| NCI | F | 70 |
| NCI | F | 80 |
| AD | M | 91 |
| AD | F | 86 |
| AD | M | 95 |
| AD | F | 77 |

**Table S2 Antibodies.**

| **Antibodies** | **Source** | **Identifier** |
| --- | --- | --- |
| WB: Western blot; IF: Immunofluorescence | | |
| SELENOK (WB) | Atlas Antibodies | HPA008196 |
| GLuA1 (WB) | Abcam | #ab31232 |
| GLuA2 (WB) | Abcam | #ab206293 |
| GLuA2 (IF) | Invitrogen | #320300 |
| GLuA3 (WB) | Invitrogen | #32-0400 |
| GluN1 (WB) | Abcam | #ab109182 |
| GluN2A (WB) | Abcam | #ab124913 |
| GluN2B (WB) | Abcam | #ab254356 |
| PSD95 (WB) | Abcam | #ab238135 |
| Synaptophysin (WB) | CST | #CST4329 |
| DHHC6 (WB) | CUSABIO | CSB-PA862029LA01HU |
| GAPDH (WB) | Proteintech | 10494-1-AP |
| β-actin (WB) | Affinity Biosciences | AF7018 |
| α-tubulin (WB) | Proteintech | 11224-1-AP |
| Calnexin (WB)(IF) | Abcam | #ab22595 |
| HA-tag (WB)(IF) | MBL | M180 |
| DDDDK-tag (WB)(IF) | MBL | M185 |
| Anti-mouse IgG HRP (WB) | CST | #7076S |
| Anti-rabbit IgG HRP (WB) | CST | #7074S |
| Anti-Rabbit Alexa Fluor 488 (IF) | Abcam | #ab150073 |
| Anti-Rabbit Alexa Fluor 568 (IF) | Abcam | #ab175470 |
| Anti-Mouse Alexa Fluor 488 (IF) | Abcam | #ab150105 |
| Anti-Mouse Alexa Fluor 568 (IF) | Abcam | #ab175472 |
| Anti-Mouse Alexa Fluor 647 (IF) | Abcam | #ab150107 |

**Table S3 Primer sequences for qPCR.**

| **Gene** | **Forward (5`- 3`)** | **Reverse (5`- 3`)** |
| --- | --- | --- |
| AMPAR1 | CCACTACATCCTCGCCAACCT | CGCCATCACCTTCACACCATC |
| AMPAR2 | TGTGAGGACTACGGCAGAAGG | CATGGTGTCGCAAGGCTTCC |
| PSD95 | TACCAAAGACCGTGCCAACG | CGGCATTGGCTGAGACATCA |
| NMDAR1 | TACAAGCGACACAAGGATGC | TCAGTGGGATGGTACTGCTG |
| NMDAR2A | CTGCTCCAGTTTGTTGGTGA | AGATGCCCGTAAGCCACA |
| NMDAR2B | GGGTTACAACCGGTGCCTA | CTTTGCCGATGGTGAAAGAT |
| Synaptophysin | CAAGGCTACGGCCAACAG | GTCTTCGTGGGCTTCACTG |
| GAPDH | ACGGCAAATTCAACGGCACAGTCA | GGTCTCGCTCCTGGAAGATGGTGAT |
| Actin | CCACCATGTACCCAGGCATT | CGGACTCATCGTACTCCTGC |
